# Recent Advances in Ophthalmic Imaging: A Decade in Review

**DOI:** 10.2174/0115734056466325260204052942

**Published:** 2026-02-17

**Authors:** Georgios D. Panos, Georgios N. Tsiropoulos, Efstratia Amaxilati, Iordanis Vagiakis, Panagiotis A.G. Konstas, Nikolaos Kozeis, Zisis Gatzioufas

**Affiliations:** 1 First Department of Ophthalmology, Ahepa University Hospital, School of Medicine, Aristotle University of Thessaloniki, Thessaloniki, Greece; 2 Department of Ophthalmology, Queen’s Medical Centre, Nottingham University Hospitals, Nottingham, UK and Division of Ophthalmology and Visual Sciences, School of Medicine, University of Nottingham, Nottingham, UK; 3 Department of Ophthalmology, School of Medicine, University of Ioannina, Ioannina, Greece; 4 Paediatric Ophthalmology Centre, Thessaloniki, Greece; 5 Department of Ophthalmology, Basel University Hospitals, Basel, Switzerland and Department of Ophthalmology, School of Medicine, University of Basel, Basel, Switzerland

**Keywords:** Ophthalmic imaging, OCT angiography, Optical coherence tomography, Adaptive optics, Confocal microscopy, Scanning laser ophthalmoscopy, Ultra-widefield imaging, Artificial Intelligence, Fluorescence lifetime imaging, Intraoperative OCT, Microperimetry, Interoperability

## 1 INTRODUCTION

The past decade has seen remarkable progress in ophthalmic imaging, reshaping both clinical decision-making and research. From dye-free angiography to cellular-level visualisation and algorithm-assisted diagnosis, contemporary platforms offer unprecedented resolution, speed, and accessibility [1, 2].

These technical advances are unfolding against a backdrop of substantial disease burden. Recent estimates suggest that vision loss remains common in high-income countries and in Eastern and Central Europe, with millions of adults living with moderate-to-severe impairment despite available therapies [3]. Age-related macular degeneration (AMD) alone affects tens of millions of people in Europe, and the absolute number is expected to rise further as populations age, even if age-specific prevalence stabilises or falls [4-6]. Imaging innovations are therefore not an academic exercise: they are critical to delivering timely diagnosis, safe surveillance, and efficient treatment at scale.

This editorial highlights key developments that have matured from promising concepts into tools with tangible impact on patient care, and outlines priorities for the next phase of translation.

## 2 TECHNOLOGY FOUNDATIONS: FASTER, DEEPER, AND RICHER

Swept-source OCT extends penetration and speeds up acquisition, improving choroidal imaging and enabling widefield volumetric scans [7]. Polarisation-sensitive and visible-light OCT add contrast for fibrotic tissue, melanin, and oxygenation-sensitive features, while phase-sensitive OCT measures nanometre-scale axial motion for functional read-outs [8, 9]. Beyond reflectivity, multispectral/hyperspectral modalities and advanced autofluorescence variants expand metabolic contrast, setting the stage for earlier disease detection [10].

Functional and metabolic imaging, including fluorescence lifetime imaging ophthalmoscopy (FLIO), retinal oximetry, and emerging photoacoustic ophthalmoscopy, aims to detect dysfunction before structural loss becomes apparent [11]. While initial work has been encouraging, particularly for FLIO and oximetry in defined cohorts, clinical validation of newer techniques such as photoacoustic ophthalmoscopy remains at an early, mainly experimental stage [12, 13].

## 3 OCT ANGIOGRAPHY: COMING OF AGE

Optical coherence tomography angiography (OCTA) has transitioned from early adoption to routine use for many indications [1]. By deriving motion contrast from repeated B-scans, OCTA maps flow in retinal and choroidal microvasculature without the need for intravenous dye. In diabetic retinopathy and age-related macular degeneration, it enables non-invasive surveillance of neovascular activity and capillary perfusion [2, 14, 15]. Compared with dye-based angiography, OCTA reduces risk, clinic time, and patient burden while providing layer-specific information; however, artefacts from motion, projection, and segmentation persist, and visualisation of deeper choroidal vessels remains challenging.

### 3.1 What’s New

Swept-source OCTA (longer wavelengths, faster acquisition) improves choriocapillaris and deep plexus visualisation; projection-resolved and slab-to-slab algorithms reduce decorrelation tails; and widefield OCTA (montage/extended field) allows pan-retinal perfusion assessment [16]. In diabetic retinopathy and age-related macular degeneration, two of the leading causes of vision loss in Europe, these tools support earlier detection and safer, more targeted monitoring of sight-threatening complications [4, 6]. Pragmatic, clinic-ready metrics, foveal avascular zone (FAZ) area and circularity, skeletonised vessel density, perfusion density, choriocapillaris flow-void mapping, and en face ellipsoid-zone integrity are increasingly used as endpoints in trials [17].

### 3.2 Unmet Needs

Consistent quantification across devices; better handling of media opacity and high myopia; harmonised flow-speed sensitivity; and normative databases stratified by age, sex, and axial length.

## 4 ULTRA-WIDEFIELD IMAGING AND THE PERIPHERAL RETINA

Ultra-widefield (UWF) platforms have shifted attention beyond the posterior pole, capturing up to 200° of the fundus in a single shot [18]. Peripheral vascular changes, tears, and inflammatory lesions are more readily documented, augmenting risk stratification and treatment planning. UWF imaging is now central to care pathways in diabetic eye disease, uveitis, and paediatric conditions [19-21].

### 4.1 Advances

True-colour, non-mydriatic confocal systems now provide better colour fidelity and peripheral autofluorescence; stereographic projection tools correct for peripheral distortion; and UWF fluorescein/ICG angiography enables pan-retinal leakage and non-perfusion mapping to guide laser [22, 23].

### 4.2 Limitations to Recognise at the Slit-lamp

Lid/lash artefacts, peripheral resolution drop-off, and montage seam effects can mimic pathology. A brief chin-positioning checklist, combined with repeat capture across different gaze positions, mitigates most issues [19].

## 5 ADAPTIVE OPTICS REACHES THE CLINIC

Adaptive optics (AO), once confined to specialised laboratories, now complements clinical instruments to achieve near-cellular resolution of photoreceptors, retinal pigment epithelium mosaics and nerve fibre bundles, as well as anterior segment microstructure [24, 25]. AO imaging has deepened the understanding of early disease mechanisms and genotype–phenotype correlations in inherited retinal disorders, and offers sensitive structural endpoints for interventional studies [26].

### 5.1 Clinical Value

Cone density and spacing maps reveal subclinical degeneration; RPE reflectance metrics track toxicity; and AO-OCT can visualise outer-segment renewal dynamics [24]. In glaucoma, AO-based bundle imaging may detect focal loss prior to RNFL thickness change [27].

### 5.2 Barriers and Solutions

Cost and limited field of view remain constraints. Emerging compact AO-SLO add-ons, automated cone-counting, and cloud-based analysis pipelines are lowering the bar for multi-centre studies [25].

## 6 ARTIFICIAL INTELLIGENCE ACROSS THE IMAGING PATHWAY

The coupling of imaging with artificial intelligence (AI) and machine learning has been the decade’s most disruptive force [28]. Deep learning systems trained on fundus photographs, OCT volumes, and UWF images now deliver performance approaching subspecialist graders for several detection tasks, and can triage high-risk cases at scale. Beyond classification, AI enables segmentation, longitudinal change detection, and prognostic modelling [28, 29].

In routine practice, AI tools are already reshaping care pathways. Autonomous and assistive systems for diabetic retinopathy screening can safely discharge low-risk patients while prioritising those needing urgent review; cloud-based platforms for OCT analysis support rapid triage of macular disease in community and emergency settings; and deep-learning models embedded in widefield imaging software quantify ischaemic burden in diabetic eye disease and uveitis [30-32]. These tools do not replace the clinician; rather, they concentrate specialist time on complex cases and longitudinal decision-making, while automating repetitive detection and measurement tasks.

To be clinically useful, AI outputs must be easy to act on at the slit-lamp or reporting workstation. In practice, this means calibrated risk scores rather than binary labels, uncertainty flags when the model is “unsure”, and simple visual explanations such as saliency overlays on fundus images or OCT B-scans [33]. Systems that highlight why a case has been flagged, and how confident the model is, are more likely to be trusted and adopted by clinicians than “black-box” predictions, even when overall accuracy is high [34]. Recent consensus frameworks for trustworthy medical AI emphasise the same themes: fairness and diversity in training data, traceability of model versions, robustness to domain shift, and explainability at the level of individual cases, not just headline metrics in a development paper [34, 35].

### 6.1 From Promising to Dependable

External validation across sites and devices, uncertainty estimation, and continuous post-deployment monitoring convert prototypes into maintainable clinical tools [36]. Federated learning and privacy-preserving analytics facilitate training on diverse datasets without raw data leaving host institutions [37].

### 6.2 Safety, Ethics, and Equity

Curate balanced datasets (including high myopia, media opacity, and multi-ethnic cohorts) publish model cards, and ensure human-in-the-loop review for outliers [38]. Workflow integration matters: AI alerts should tell the clinician clearly what the concern is, how confident the system is, and where to look in the image, and they should appear naturally within existing reporting software rather than as a separate screen.

## 7 PORTABILITY AND POINT-OF-CARE IMAGING

Handheld and portable devices have broadened access to high-quality imaging in theatres, wards, emergency departments, neonatal units, and community settings [39, 40]. These platforms particularly benefit paediatric and bed-bound patients, and reduce barriers in low-resource environments. While image quality can lag that of benchtop systems, and battery life/durability are practical concerns, rapid improvements in sensors, optics, and onboard processing are closing the gaps.

### 7.1 What’s Changing

Smartphone-assisted fundus and slit-lamp adaptors, handheld OCT for NICU and theatre, and portable confocal microscopy enable decision-making at the bedside [41]. Real-time AI triage, on-device or in the cloud, can prioritise referrals without additional clinic visits.

### 7.2 Implementation Tips

Train super-users, standardise capture protocols, and use secure, automated image transfer to the electronic record to avoid ‘orphan’ images.

## 8 INTRAOPERATIVE AND DIGITALLY ASSISTED SURGERY

Intraoperative OCT integrated into microscopes provides real-time feedback during macular surgery, DMEK/DALK, and complex retinal detachment repair, confirming cleavage planes, graft/flap position, and subretinal fluid dynamics [42]. Heads-up 3-D visualisation with digital overlays aligns pre-operative imaging with the surgical field and supports teaching and tele-mentoring.

## 9 STRUCTURE–FUNCTION INTEGRATION

Linking imaging with microperimetry, contrast sensitivity, dark adaptation, and objective electrophysiology turns structural maps into functional risk models [43]. Co-registered change maps help decide when to treat, when to switch therapy, and how to stratify trial cohorts.

## 10 DATA QUALITY, GOVERNANCE, AND INTEROPERABILITY

To get the most value from these images, three elements are crucial: reliable image quality checks, standardised file formats that work across vendors, and straightforward integration with routine electronic records. Data stewardship should cover consent for secondary use, lineage tracking, and retention schedules. Interoperability with electronic records and registries unlocks real-world evidence.

## 11 EDUCATION AND WORKFORCE

New imaging brings new skills. Short, case-based curricula for imaging technicians and clinicians, covering capture, artefact recognition, and interpretation, are essential. Credentialing pathways and simulation with synthetic data will speed safe uptake.

## CONCLUSION

Ophthalmic imaging has entered a mature phase in which resolution, field of view, speed, and analytical capability reinforce one another. Realising the full benefits for patients will depend less on incremental hardware gains and more on integration: integrating modalities, integrating quantitative metrics into trials and clinics, and integrating AI within safe, ethical, and efficient care pathways. The coming decade should focus on turning exquisite images into actionable, equitable information that protects sight.

## LIST OF ABBREVIATIONS

AMD: Age-related Macular Degeneration
FLIO: Fluorescence Lifetime Imaging Ophthalmoscopy
OCTA: Optical Coherence Tomography Angiography
FAZ: Foveal Avascular Zone
AO: Adaptive Optics
